# Analysis of factors influencing fall risk among elderly people in rural of China

**DOI:** 10.1038/s41598-024-60430-x

**Published:** 2024-04-27

**Authors:** Yaodong Zhao, Dan Xie, Chi Zhang, Haibo Wang, Beibei Zhang, Song Liu, Min Li, Guimei Chen, Hong Ding

**Affiliations:** 1 Department of Health Service Management, School of Health Management, Anhui Medical University, Anhui, China; 2 School of Health Management, Anhui Medical University, No. 81 Meishan Road, Hefei, 230032 Anhui China

**Keywords:** Rural areas of China, Senior citizen, Risk of falls, Influencing factors, Falls, Health occupations, Risk factors

## Abstract

Falls can cause serious health problems in the elderly. China is gradually entering a moderately aging society. In rural areas of China, the elderly are at a higher risk of falling. This study aims to explore and analyze the factors affecting the fall risk of elderly people in rural areas of China, and provide theoretical basis for reducing the fall risk of elderly people. M County, Anhui Province, China was selected as the survey site by the typical field sampling method, and the elderly people in rural areas were selected as the research objects. A total of 1187 people were investigated. Mann–Whitney U test and Kruskal–Wallis H test were used for univariate analysis, and multiple linear regression was used for multivariate analysis. Chronic diseases, multimorbidity, daily living ability, mental health, working status and family doctors are the factors that influence falls among elderly people in rural areas of China (P < 0.05, Adjusted R^2^ = 0.395). The falls risk of the elderly in rural areas of China is influenced by multiple factors. Therefore, comprehensive measures should be taken to reduce the fall risk by comprehensively evaluating the influencing factors.

## Introduction

According to the results of China's seventh census, the total number of the elderly aged 60 and above reached 264 million, accounting for 18.70% of the total population of the country, which increased by 5.44% compared with the China's sixth census. The aging degree of the population will be further deepened and gradually enter a moderately aging society^1^. Approximately one-third of the elderly population in China experience at least one fall per year. Falling, as a significant determinant impacting the well-being of older adults, has become increasingly prominent with the progression of aging^2,3^.

The World Health Organization defines a fall as a" sudden, not intentional, and unexpected movement from orthostatic position, from seat to position, or from clinical position"^4^. Currently, falls are recognized as a major public health problem worldwide and the number one global health problem^5^. Falling will cause serious health problems for the elderly, not only bring heavy burden to the family and society, but also increase the consumption of medical resources^6^. There are many factors affecting falls risk in the elderly, which are mainly divided into intrinsic and extrinsic factors. Intrinsic factors are internal factors such as age, gender, physical health, mental health and weak gait, and the extrinsic factors mainly include behavior, lifestyle, living environment and living conditions, which may influence the occurrence of falls risk^7–9^. There may be differences in fall risk factors in different regions. Elderly people often fall not because of any one factor, but the result of multiple factors^10,11^. Therefore, it is very important to identify the factors that affect the fall risk of the elderly and reduce the fall risk of the elderly.

With the increase of age, the physiological indexes and functions of the elderly population gradually decline, and the fall risk and street damage rate also increase^12,13^. Gender is also an important factor affecting the fall risk of the elderly. Due to the faster bone loss of the elderly after menopause, and the fact that women generally bear more housework in the family, the fall risk of women is often higher than that of men in some studies^14,15^. Grip strength can reflect the muscle strength of the elderly and is associated with the risk of falls in the elderly, and studies have pointed out that elderly people with low grip strength have a higher risk of falls^16,17^. The engagement in physical activity can contribute to the preservation of physical function and mobility among older adults, while a certain level of physical activity can effectively mitigate their susceptibility to falls^18^. Older adults with mental health conditions may face an elevated risk of experiencing falls, and this risk is further amplified among older adults who are prescribed medications for their mental illness^19^.

Long-term alcohol consumption has detrimental effects on the physical health and cognitive function of older adults^20^, as well as their gait and reaction abilities. A meta-analysis reveals that elderly individuals who consume alcohol have an increased risk of falling^21^. Research on living environments and fall risks indicates that factors such as uneven ground surfaces, wet conditions, inadequate lighting, and cluttered pathways also contribute to the fall risk among older adults^22,23^. Rural areas often lack age-appropriate infrastructure and design, making elderly residents more susceptible to falls and higher fall risks^24,25^. Additionally, many elderly individuals residing in rural areas are unaware of their own susceptibility to falls^26^.

Falls have been identified as the primary cause of fatal injuries among elderly individuals in China^27^. It is clearly pointed out in the outline of Healthy China 2030 plan that the elderly fall prevention intervention is an important part of the health promotion of the elderly in China^28^ After conducting a comprehensive review of existing literature on fall risk among elderly individuals in rural areas of China, we have found a recent lack of research on factors specifically related to this particular risk. This study aims to explore and analyze the factors affecting the fall risk of elderly people in rural areas of China, and provide theoretical basis for reducing the fall risk of elderly people.

## Methods

A cross-sectional survey was conducted in M County, Anhui Province, central China from July to September 2021. M County is one of the pilot counties for the construction of compact county medical community in Anhui province. The local has a perfect medical and health service system, and the grass-roots staff has a high degree of cooperation, which provides good conditions for this study.

Two townships in M County, Anhui Province, China were randomly selected, and 5 villages were randomly selected in each township. All elderly people in these 10 villages were selected as survey objects. At present, the definition of the elderly at home and abroad can be roughly divided into two kinds: one defines the age of the elderly as 60 years old and above, the other defines the age of the elderly as 65 years old and above. Since the World Congress on Aging and the Law of the People's Republic of China on the Protection of the Rights and Interests of the Elderly both define the elderly as those aged 60 and above^29^, the object of this study is the elderly aged 60 years and above. The inclusion criteria also require a minimum residency duration of one year. Exclusion criteria encompass individuals who are critically ill and unable to actively participate in the study, as well as those with cognitive and communication impairments that hinder normal communication abilities. The investigators were postgraduates from the School of Health Management at Anhui Medical University who successfully completed comprehensive training. The study adhered to the principles outlined in the Declaration of Helsinki, ensuring informed consent, privacy protection, and voluntary participation during field investigations.

On the basis of referring to relevant literature, a questionnaire was designed based on the characteristics and living conditions of the elderly population in rural areas of China. The questionnaire included socio-demographic characteristics, personal health, ability to perform daily living and sleep quality. Personal and social characteristics included gender (male/female), age (60–69 years; 70–79 years; ≥ 80 years old), education level (primary school and below/secondary school and above), marital status (married; divorce; the death of a spouse; other), residential status (living alone/not living alone), working status (employed/not employed), main source of income (Labor income/non-labor income, if the main source of income is labor, defined as income from labor, if the main source of income is government subsidies or child support, defined as unearned income), smoking (yes/no) and alcohol consumption (yes/no); Personal health conditions include height, weight, grip strength, blood pressure, chronic diseases (yes/no), multimorbidity (yes/no, two or more chronic diseases are considered yes, one or less is considered no) and mental health; According to the scale measurement, the ability of daily living was divided into needing help and not needing help. Sleep quality was measured according to the scale to determine whether the elderly had sleep disorders.. Before each survey, investigators composed of uniformly trained graduate students from Anhui Medical University and doctors from local township health centers visited the survey subjects to dictate the research objectives and procedures, and then conducted face-to-face interviews. A total of 1200 questionnaires were distributed in this survey, and 13 questionnaires that could not be used due to multiple selection and missing selection were excluded. A total of 1187 effective questionnaires were collected, with effective recovery of 98.92% (1187/1200).

### Measurement of fall risk

Morse Fall Risk Assessment Scale (MFS): Developed by American scholar Morse et al.^30^ in 1989, this scale was originally adapted by Chinese scholar Zhou^31^ to measure the fall risk of hospitalized elderly patients, and is now widely used to measure the fall risk of elderly people in communities^32–35^. The scale consisted of six items, including history of falls, diagnosis of more than one disease, use of assistive devices while walking, use of intravenous fluids or medication, gait/movement and mental state. Each item was scored on a scale ranging from 0 to 25 points, with a total score of 125. The higher the score, the greater the risk of falls.

### Measurement of mental health

The 12-item general health questionnaire (GHQ-12): The 12-item General Health Questionnaire, GHQ-12: This scale is suitable for the mental health assessment of the elderly population^36^. There are 12 items in total, and the answers for each item include four levels: none at all, the same as usual, more than usual and much more than usual. According to the 0-0-1-1 scoring method, 0 points are recorded when the survey subjects choose 1 or 2, and 1 point is recorded when they choose 3 or 4. The higher the score, the worse the psychological condition, higher than 3 points as a tendency to psychological problems, lower than 3 points as normal.

### Measurement of daily living ability

Barthel Index (BI): The scale is a widely used scale^37^, which includes 10 items including eating, bathing, grooming (including washing face, brushing teeth, shaving and combing hair), dressing (including tying shoelaces, etc.), stool control, urination control, going to the toilet (including wearing and undressing and using toilet paper), transfer, flat walking and going up and down stairs, with a total score of 100. A score of 100 indicates that the respondents can complete the activities of daily living independently, while a score less than 100 indicates that the respondents need to rely on others for their activities of daily living^38^.

### Measurement of sleep quality

Pittsburgh sleep quality index (PSQI): This scale was compiled by psychiatrist Buysse et al., University of Pittsburgh^39^, and has good reliability and validity in the rural elderly population^40^. The scale consists of seven dimensions: sleep quality, time to fall asleep, sleep time, sleep efficiency, sleep disorders, hypnotic drugs and daytime dysfunction. Each dimension is 0–3 points, and the total score is the sum of all PSQI dimensions. The higher the total score, the worse the sleep quality, and when the total score is greater than 7, the sleep disorder is considered^41^.

### Statistical analysis

EpiData 3.1 software was used for data entry, and SPSS 26.0 software was used for data correction and statistical analysis. After normality test, there was a skewed distribution of fall risk, so median and quartile M (P_25_, P_75_) were used for statistical description. Mann–Whitney U test and Kruskal–Wallis H test were used for univariate analysis during the analysis. Statistically significant variables in univariate analysis were included in multiple linear regression to analyze the influencing factors of fall risk. P < 0.05 was considered statistically significant.

### Ethics approval and consent to participate

This study was conducted in accordance with the Declaration of Helsinki and was approved by the Research Ethics Committee of Anhui Medical University. All participants were fully informed about the study purpose and methods. Before conducting the survey, explain the purpose and procedures of the research to all interviewees, and ensure that all interviewees have informed consent to this research. For the illiterate interviewees, the informed consent of the guardian was also obtained.

### Contribution to the field

Falls can cause serious health problems in the elderly. China is gradually entering a moderately aging society, and in China's rural areas, the elderly fall rate is higher. There have been many studies on the factors affecting the fall risk of the elderly, but the research results may be different due to the differences in economic and social conditions, medical security services, residents' health awareness and other aspects in different regions. The purpose of this study was to explore the factors affecting the fall risk of the elderly in rural areas of Anhui Province, China, and to find out the effects of two factors, contracted family doctors and working status, on the fall risk of the elderly, providing a theoretical basis for reducing the fall risk of the elderly and further taking preventive measures.

## Results

### Basic information of survey objects

Table 1 describes the specific situation of falls among elderly people in rural areas. Among the elderly surveyed, 198 had fallen in the past 3 months, with a fall incidence of 16.88%. Among them, the age group between 60–69 experienced an incidence rate of falls at 17.07%. Furthermore, males had an incidence rate of falls at 16.98%, while females had a slightly lower rate at 16.39%.

**Table 1:** Incidence of falls in rural elderly people.

Variable	Fall		Total
	Yes	No	
Total	198 (16.88%)	989 (83.12%)	1187
Age (years)			
60–69	70 (16.88%)	340 (83.12%)	410
70–79	97 (17.07%)	485 (82.93%)	582
≥ 80	31 (16.67%)	163 (83.33%)	194
Gender			
Male	99 (16.98%)	484 (83.02%)	583
Female	99 (16.39%)	505 (83.61%)	604

Table 2 describes the general demographic characteristics of the respondents. There are 583 (49.12%) males and 604 (50.88%) females among 1187 elderly people in rural areas. 582 people (49.03%) aged 70–79 are the largest.

**Table 2:** General characteristics of survey subjects and single factor test results of factors affecting fall risk of rural elderly (N = 1187).

Variable	n (%)	M* (P_25_, P_75_)	Z/H	P
Gender			2.974	0.030
Male	583 (49.12)	35 (15–35)		
Female	604 (50.88)	35 (20–45)		
Age (years)			10.790	0.050
60–69	410 (34.54)	35 (15–35)		
70–79	582 (49.03)	35 (20–35)		
≥ 80	195 (16.43)	35 (20–50)		
Education level			−3.939	< 0.001
Primary and below	970 (81.72)	35 (20–45)		
Junior and above	217 (18.28)	35 (20–35)		
Married status			5.378	0.146
Married	835 (70.35)	35 (20–35)		
Divorced	8 (0.67)	25 (35–47.5)		
Widowed	293 (24.68)	35 (20–35)		
Other	51 (4.30)	35 (30–45)		
Living style			0.133	0.936
Living alone	254 (21.40)	35 (20–35)		
Living with spouse	631 (53.16)	35 (20–35)		
Living with others	302 (25.44)	35 (15–45)		
Working status			−7.335	< 0.001
Work	959 (80.79)	35 (20–35)		
Don’t work	228 (19.21)	35 (35–60)		
The main source of income			−5.543	< 0.001
Income from labor	603 (50.80)	35 (15–35)		
Unearned income	584 (49.20)	35 (20–45)		
BMI			111.953	0.003
Low body weight	54 (4.55)	35 (15–35)		
Normal weight	488 (41.11)	35 (15–35)		
Overweight	645 (54.34)	35 (20–45)		
Grip strength			−5.037	< 0.001
Normal	947 (79.44)	35 (25–50)		
On the low side	240 (20.56)	35 (20–45)		
Pulse pressure difference			3.174	0.002
Normal	834 (70.26)	35 (15–35)		
Exceed the standard	353 (29.74)	35 (20–45)		
Chronic diseases			17.977	< 0.001
Yes	917 (77.25)	35 (35–45)		
No	270 (22.75)	0 (0–25)		
Multimorbidity			−15.259	< 0.001
Yes	518 (43.64)	35 (35–50)		
No	669 (56.36)	20 (0–35)		
Daily living ability			−0.897	< 0.001
Without help	206 (17.35)	35 (15–35)		
Need help	981 (82.65)	35 (35–60)		
Sleep quality			−4.162	< 0.001
Good	403 (33.95)	35 (0–35)		
Bad	784 (66.05)	35 (20–45)		
Mental health			−5.547	< 0.001
Good	1020 (85.93)	35 (20–35)		
Bad	167 (14.07)	35 (35–55)		
Smoking			−4.612	< 0.001
No	230 (19.38)	35 (0–35)		
Yes	957 (80.62)	35 (20–45)		
Drink alcohol			−5.240	< 0.001
No	287 (24.18)	30 (0–35)		
Yes	900 (75.82)	35 (20–45)		
Family doctor			−3.110	< 0.001
No	813 (68.49)	35 (20–35)		
Yes	374 (31.51)	35 (20–45)		

BMI: Body Mass Index.

M*: median.

### Linear regression analysis of fall risk among elderly people in rural areas

Table 3 describes the results of multiple linear regression. The results show that chronic disease, multimorbidity, ability to live daily, mental health, work status and family doctor are associated with fall risk.

**Table 3:** Linear regression analysis of influential factors of fall risk.

Variable	β	SE	Beta	t	P	VIF
Constant	13.035	3.596		3.625	< 0.001	
Working status	−6.613	1.33	−0.128	−4.971	< 0.001	1.298
Multimorbidity	7.462	1.086	0.182	6.871	< 0.001	1.371
Daily living ability	8.469	1.287	0.158	6.581	< 0.001	1.122
Mental health	3.963	1.387	0.068	2.858	0.004	1.099
Family doctor	2.112	1.014	0.048	2.083	0.037	1.049
Chronic diseases	19.158	1.267	0.398	15.127	< 0.001	1.360

Dependent variable: Fall risk.

Adjusted R^2^ = 0.395.

## Discussion

In this survey, the incidence of falls among the elderly in rural areas of China is 16.88%, which is lower than the result of the analysis of Mate Wang Xiaojun et al. in 2020 (the incidence of falls among the elderly in rural communities is 21.5%)^42^. The incidence of falls among the elderly aged 60 and above in rural areas was 8.6% higher than that of Qi Shige et al. in 2011^43^, which was similar to the results of Zhang L et al. in 2019 on 8840 elderly in urban areas and 7953 elderly in rural areas in 20 provinces (urban: 14% and rural: 17%)^44^. The results of each study may be different due to different factors such as sample size, survey time, population characteristics and region. Multiple linear regression analysis indicated that family doctor, chronic disease, multimorbidity, daily living ability, working status and mental health were the factors affecting the fall risk, and the independent variables in this analysis could explain 39.5% of the change in fall risk.

Elderly people with chronic diseases and multimorbidity have a higher risk of falling, which is consistent with previous studies^45–48^. Chronic diseases will cause damage to the health of the elderly, and, the elderly with one chronic disease will often develop a variety of chronic diseases. The damage to the health of the elderly is usually accompanied by product effect, which leads to further decline of the health status of the elderly and higher risk of falling^49,50^.

Rural elderly with psychological problems are at risk of falling^51,52^. These older people with mental are.

prone to loneliness, anxiety, depression and other negative emotions, which increase the risk of falling. Moreover, mental illness will increase the risk of disease in the elderly^53^, damage the health status of the elderly, and then increase the risk of falling.

Studies have shown that elderly people who are not dependent in daily living activities have a lower risk of falling, which is consistent with previous studies^54^. In terms of working status, working elderly people have a lower risk of falling than those who do not work. In China, farming civilization determines the characteristics of Chinese culture, and farming is the main feature in China's rural areas. The survey shows that there are more left-behind elderly people in China's rural areas, and more than half of them need to rely on their own labor to support themselves^55^. Elderly people who work are exposed to high risk of falling and theoretically have a higher risk of falling. In the current study, however, older people who worked had greater ability to perform activities of daily living;.. Secondly, previous studies have shown that the labor participation rate of residents will increase with the increase of health status^56^, and the health status of the elderly working is better than that of the elderly not working. Thirdly, physical exercise is an important factor affecting the physical and mental health of the elderly, moderate physical exercise can play an important role in improving the physical and mental health of the elderly^57–60^, while the situation of physical exercise in rural areas is poor^56^, labor can replace physical exercise to a certain extent, and contribute to the physical and mental health of the elderly. The health of the working elderly is relatively better. Older people who work are at lower risk of falls due to their greater capacity for activities of daily living and better health.

Unexpectedly, elderly people who signed up with family doctors have a higher risk of falling. Family doctor contract service is a personalized service package with general practitioners as the core, residents' voluntary contract as the basis, and residents' health conditions and needs as the guidance. It provides services in the form of team service, which is one of the important tasks of China's medical and health system reform. Family doctors can provide preventive, health, medical and rehabilitation services to residents, but this study found that elderly people who signed up with family doctors had a higher risk of falling. On the one hand, although family doctors have made preliminary progress in China, there is still a certain gap between urban and rural areas. In rural areas, there are insufficient number of family doctors, low educational and professional level, which greatly affects the quality of family doctor services^61^. On the other hand, the study of Zhang Cuiping et al. shows that in China, the worse the health status of residents, the higher the signing rate of family doctors^62,63^, and the elderly with worse health status are more likely to sign up for family doctor services. Therefore, the group of older people who signed up with family doctors in rural areas have poorer health and are not sufficiently improved by family doctor services, leading to a higher risk of falls.

## Conclusion

To sum up, the fall risk of the elderly in rural areas of China is related to chronic diseases, multimorbidity, grip strength and other factors, and the fall risk of the elderly is the result of multiple influencing factors. When judging the factors affecting the elderly fall risk, it is necessary to evaluate them in various aspects, so that targeted intervention measures can be taken to reduce the fall risk and reduce the occurrence of falls, so as to enhance the happiness of the elderly and help the development strategy of "Healthy China".

### Advantages and limitations

Advantages: First, the effective response rate of this study is 98.92% (1187/1200), as we all know, studies with higher effective response rates were more reliable. Secondly, an internationally recognized measurement questionnaire was used to measure the objects objectively. In addition, this is the first time to use multiple linear regression to study the factors affecting the fall risk of the elderly in rural Anhui Province, and a new situation has been found that the elderly who work have a lower risk of falling and the elderly who contract with family doctors have a higher risk of falling.

However, this study also has the following limitations: First, there was a lack of investigation into frailty, fear of falling, dizziness, and the living environment of the study subjects, which may be important factors affecting fall risk and were not included in this study. Secondly, the survey objects of this study only cover rural areas of M County in Anhui Province. Due to the differences in economic development and cultural background in different regions, the scalability of the results of this study is limited.

## Data Availability

The datasets generated during the study are not publicly available due to an ethical restriction but are available from the corresponding author on reasonable request.
